# Supplementary material for: Efficacy of vonoprazan against bleeding from endoscopic submucosal dissection-induced gastric ulcers under antithrombotic medication: A cross-design synthesis of randomized and observational studies
Source: PLoS One. 2021 Dec 23;16(12):e0261703. doi: 10.1371/journal.pone.0261703 (PMC8699580; doi:10.1371/journal.pone.0261703)
Supplement: S1 Table — (PDF) [file pone.0261703.s001.pdf]

Supporting information

S1 Table. Example of full electronic search strategy used for the online database

Data source		Number of hits
PubMed (-2020 November)	1. vonoprazan	269
	2. Takecab	5
	3. TAK-438	185
	4. potassium-competitive inhibitor	201
	5. ESD	5038
	6. endoscopic submucosal dissection	4638
	7. 1 OR 2 OR 3 OR 4	338
	8. 5 OR 6	6724
	9. (1 OR 2 OR 3) AND (4 OR 5 OR 6)	31
