# Supplementary material for: Efficacy of vonoprazan against bleeding from endoscopic submucosal dissection-induced gastric ulcers under antithrombotic medication: A cross-design synthesis of randomized and observational studies
Source: PLoS One. 2021 Dec 23;16(12):e0261703. doi: 10.1371/journal.pone.0261703 (PMC8699580; doi:10.1371/journal.pone.0261703)
Supplement: S1 Appendix — (PDF) [file pone.0261703.s002.pdf]

# Supporting information

## S1 Appendix. The CDS estimator

In studies of antithrombotic drugs, CDS uses data from an RCT and an observational study to estimate the Population Average Treatment Effect (PATE) $\Delta$ for the overall treated patients, including both patients taking and not taking antithrombotic drugs. Let $A$ denote a variable that shows patients selection for the RCT. Patients not taking antithrombotic drugs included in the RCT are $A = 0$, patients taking antithrombotic drugs excluded from the RCT are $A = 1$, and the Subpopulation Average Treatment Effect (SPATE) of patients taking antithrombotic drugs is $\Delta(A = 0)$ and that of patients not taking antithrombotic drugs is $\Delta(A = 1)$. Then $\Delta$ becomes

$$\Delta = p_0\Delta(A = 0) + p_1\Delta(A = 1). \tag{1}$$

where $p_0$ and $p_1$ are respectively the percentages of the overall treated patients who are not taking antithrombotic drugs and those who are taking antithrombotic drugs, respectively.
Here, assuming that the RCT was conducted in patients taking and not taking antithrombotic drugs, the estimators of $\Delta(A = 0)$ and $\Delta(A = 1)$ using the results of the RCT becomes $\hat{\Delta}_R(A = 0)$ and $\hat{\Delta}_R(A = 1)$. Let the naive estimator of $\Delta$ be

$$\hat{\Delta} = p_0\hat{\Delta}_R(A = 0) + p_1\hat{\Delta}_R(A = 1). \tag{2}$$

Since patients not taking antithrombotic drugs are included in the RCT, $\hat{\Delta}_R(A = 0)$ can be calculated from the RCT results. However, it is difficult to conduct the RCT in patients taking antithrombotic drugs, so data from an observational study will be used for $\hat{\Delta}_R(A = 1)$. Here, if the difference between the SPATE of patients taking antithrombotic drugs and that of patients not taking antithrombotic drugs is $d$, then $d$ becomes

$$d = \Delta(A = 1) - \Delta(A = 0). \tag{3}$$

Furthermore, let $\hat{\Delta}_O(A = 0)$ and $\hat{\Delta}_O(A = 1)$ be the estimators of $\Delta(A = 0)$ and $\Delta(A = 1)$, respectively, from the results of the observational study, $\hat{d}$, in which case the estimator of $d$ becomes

$$\hat{d} = \hat{\Delta}_O(A = 1) - \hat{\Delta}_O(A = 0). \tag{4}$$

Since patients of the observational study is not randomized, $\hat{\Delta}_O(A = 1)$ and $\hat{\Delta}_O(A = 0)$ can be biased due to treatment selection error. However, Kaizer[18] showed that if the biases of $\hat{\Delta}_O(A = 1)$ and $\hat{\Delta}_O(A = 0)$ are equal, then the bias of $\hat{d}$(defined as the former value minus the latter value) is zero, is zero, and thus the following approximately holds:

$$\hat{d} = \hat{\Delta}_O(A = 1) - \hat{\Delta}_O(A = 0) = \hat{\Delta}_R(A = 1) - \hat{\Delta}_R(A = 0). \tag{5}$$

From this, $\hat{\Delta}_R(A = 1)$ can be expressed by the following equation:

$$\hat{\Delta}_R(A = 1) = \hat{\Delta}_R(A = 0) + [\hat{\Delta}_O(A = 1) - \hat{\Delta}_O(A = 0)]. \tag{6}$$

Furthermore, $\hat{\Delta}$ can be obtained from the following equation:

$$\hat{\Delta} = \hat{\Delta}_R(A = 0) + \hat{p_1}[\hat{\Delta}_O(A = 1) - \hat{\Delta}_O(A = 0)], \tag{7}$$

Where $\hat{p_1}$ is the estimator of $p_1$, which is defined as $\hat{p_1} = n_1/n$ using the sample size n of the overall treated patients and the sample size $n_1$ of patients taking the antithrombotic drugs in the observational study.
