# Supplementary material for: Efficacy of vonoprazan against bleeding from endoscopic submucosal dissection-induced gastric ulcers under antithrombotic medication: A cross-design synthesis of randomized and observational studies
Source: PLoS One. 2021 Dec 23;16(12):e0261703. doi: 10.1371/journal.pone.0261703 (PMC8699580; doi:10.1371/journal.pone.0261703)
Supplement: S2 Appendix — (PDF) [file pone.0261703.s003.pdf]

Supporting information

S2 Appendix. Sufficient assumptions that the CDS estimator is unbiased

As mentioned, the CDS estimator is unbiased if the assumption that the expected treatment error for stratified estimators from the observational study is constant across strata. While the bias due to measurable confounders can be adjusted, that due to unmeasurable confounders cannot. Kaizer[18] introduced the following condition to ensure that the CDS estimator is unbiased: *'confounding due to inclusion criteria and other variables are separate in the sense that X is independent of U both conditionally and unconditionally on treatment($X \perp U \mid T$ and $X \perp U$) and the average outcome does not depend on any interaction between X and U'*, where X is the inclusion criterion, U is the unobserved variable, and T is treatment. Furthermore, it is not possible to examine whether this assumption is met by statistical methods and it should therefore be examined based on clinical findings.

In the observational study data analysed here, confounding adjustment by matching was performed for measurable confounders that may be involved in the outcome, namely post-ESD bleeding. However, an unmeasured confounding factor is the skill of the ESD practitioner in performing the procedure. The observational study was conducted from 2005 to 2014 (before the launch of vonoprazan) in the PPI group, and from 2014 to 2018 (after the launch of vonoprazan) in the vonoprazan group, so physicians' proficiency in the technique may have increased over time. This suggests that proficiency may have varied according to treatment drug (vonoprazan or PPI). Therefore, physician proficiency is considered to be an unobservable confounding factor.

In this study, the conditions in which the CDS estimator is unbiased for the unmeasured confounding factor of physician proficiency are as follows. First, physician proficiency is independent of antithrombotic drug use. Second, the effect of physician proficiency on post-ESD bleeding is independent of antithrombotic drug use and vonoprazan treatment effect. All ESDs performed in the observational study were performed by one of two physicians on the same team, and physicians in charge did not change depending on whether the patient was receiving antithrombotic drugs or not. In addition, patients receiving antithrombotic drugs are more prone to bleeding, even from minor injuries, and vonoprazan has also been shown to be effective against more severe gastric acid-related mucosal injury. Therefore, the risk of bleeding from taking antithrombotic drugs and the treatment effect of vonoprazan may remain the same regardless of whether lesser physician skill results in the formation of a more bleeding-prone post-ESD ulcer. In summary, the unmeasured confounder of physician proficiency that was considered in this study satisfies the condition of the unbiased CDS estimator.
