# Supplementary material for: Efficacy of vonoprazan against bleeding from endoscopic submucosal dissection-induced gastric ulcers under antithrombotic medication: A cross-design synthesis of randomized and observational studies
Source: PLoS One. 2021 Dec 23;16(12):e0261703. doi: 10.1371/journal.pone.0261703 (PMC8699580; doi:10.1371/journal.pone.0261703)
Supplement: Attachment — Submitted filename: Response_to_Reviewers.docx [file pone.0261703.s004.docx]

We wish to express our appreciation to the Reviewer for his or her insightful comments, which have helped us significantly improve the paper.

We have revised the manuscript in accordance with the reviewers’ comments. The page and line numbers shown in this letter are in accordance with the revised manuscript.

**Response to Journal requirements:**

**Comment 1:**

Please ensure that your manuscript meets PLOS ONE's style requirements, including those for file naming. The PLOS ONE style templates can be found at

https://journals.plos.org/plosone/s/file?id=wjVg/PLOSOne_formatting_sample_main_body.pdf and

https://journals.plos.org/plosone/s/file?id=ba62/PLOSOne_formatting_sample_title_authors_affiliations.pdf.

**Response:**

Thank you for pointing this out. I have modified the overall style of the manuscript. The title, authors, and affiliations have been modified to match the journal style (Page1). Author Contributions have been added and listed in the section (Page 14, Lines 506–509).

**Comment 2**:

Please ensure you have provided the full electronic search strategy for at least one database.

**Response:**

In accordance with the reviewers’ comments, we have added a table of complete electronic search strategies for different databases as S1 Figure, summarizing the results for the PubMed database among the three databases.

We have corrected the following text in the Methods section (Page 3, Line 73–75).

“An example of a full electronic search strategy used for the online database is shown in S1 Table and a flow diagram for assessing the studies is shown in Fig 1.”

**Comment 3**:

We note that the grant information you provided in the ‘Funding Information’ and ‘Financial Disclosure’ sections do not match.

When you resubmit, please ensure that you provide the correct grant numbers for the awards you received for your study in the ‘Funding Information’ section.

**Response:**

We apologize for the lack of confirmation regarding the funding information. We have included financial disclosure in the cover letter as well as our response to the following comment 4.

**Comment 4:**

Thank you for stating the following in the Acknowledgments/ Funding Section of your manuscript:

“This study was funded by AMED under Grant Numbers JP19lk0201061 and JP20lk0201061.”

We note that you have provided funding information that is not currently declared in your Funding Statement. However, funding information should not appear in the Acknowledgments section or other areas of your manuscript. We will only publish funding information present in the Funding Statement section of the online submission form.

Please remove any funding-related text from the manuscript and let us know how you would like to update your Funding Statement. Currently, your Funding Statement reads as follows:

“The funders had no role in study design, data collection and analysis, decision to publish, or preparation of the manuscript.”

Please include your amended statements within your cover letter; we will change the online submission form on your behalf.

**Response:**

The correct Financial Disclosure is: “This study was funded by AMED under Grant Numbers JP19lk0201061 and JP20lk0201061 and 21lk0201702.” I have written this in the cover letter. We sincerely regret this error.

**Comment 5**:

In your Data Availability statement, you have not specified where the minimal data set underlying the results described in your manuscript can be found. PLOS defines a study's minimal data set as the underlying data used to reach the conclusions drawn in the manuscript and any additional data required to replicate the reported study findings in their entirety. All PLOS journals require that the minimal data set be made fully available. For more information about our data policy, please see http://journals.plos.org/plosone/s/data-availability.

Upon re-submitting your revised manuscript, please upload your study’s minimal underlying data set as either Supporting Information files or to a stable, public repository and include the relevant URLs, DOIs, or accession numbers within your revised cover letter. For a list of acceptable repositories, please see http://journals.plos.org/plosone/s/data-availability#loc-recommended-repositories. Any potentially identifying patient information must be fully anonymized.

Important: If there are ethical or legal restrictions to sharing your data publicly, please explain these restrictions in detail. Please see our guidelines for more information on what we consider unacceptable restrictions to publicly sharing data: http://journals.plos.org/plosone/s/data-availability#loc-unacceptable-data-access-restrictions. Note that it is not acceptable for the authors to be the sole named individuals responsible for ensuring data access.

We will update your Data Availability statement to reflect the information you provide in your cover letter.

**Response:**

Given the use of human research participants’ data in this study, disclosing further information may reveal sensitive patient information and cannot be shared in the open domain. Data is available from the Ethics Committee of Kagawa Prefectural Central Hospital (contact via their website; http://www.chp-kagawa.jp/) for researchers who meet the criteria to access confidential data. I have mentioned this in the cover letter.

**Response to** **Reviewers' comments:**

**Reviewer #1**

**Comment 1:** From table 5, the percentage of combination of antiplatelet and anticoagulant (warfarin) was obviously higher in PPI group (0% vs. 57%). This maybe the factor contributing higher rebleeding rate in PPI group. Please discuss on this issue.

**Response:**

We have added the following sentences to the Discussion section (Page 9, Lines 260–264).

“The post-ESD bleeding rate with the combined use of an antiplatelet and anticoagulant (warfarin or DOAC), which are presumed to pose the highest risk of bleeding, was lower in the vonoprazan group than in the PPI group (20.0% vs. 62.5%). The superiority of vonoprazan over PPI in preventing post-ESD bleeding may, therefore, be more pronounced in patients at a high risk of bleeding.”

**Comment 2:**

In discussion part, the author stated that “In patients taking antithrombotic drugs, vonoprazan was expected to reduce post-ESD bleeding to a greater extent than PPIs, and the observational study by Kagawa et al. [7] suggested that vonoprazan reduced post-ESD bleeding in patients taking antithrombotic drugs. However, these results were inconsistent with those of the subsequent RCT by Hamada et al. [8], which led us to perform the present study.” In fact, the study by Hamada excluded the patients taking antithrombotic drugs, thus the results were different from previous observational study as they assessed the patients in different group. Please consider re-write.

**Response:**

We appreciate your comment. We have deleted the following sentences: “In patients taking antithrombotic drugs, vonoprazan was expected to reduce post-ESD bleeding to a greater extent than PPIs, and the observational study by Kagawa et al. [7] suggested that vonoprazan reduced post-ESD bleeding in patients taking antithrombotic drugs.”.

We have adapted it as follows (Page 8, Line 224–227).

“An observational study by Kagawa et al.[9] suggested that vonoprazan significantly reduced post-ESD bleeding compared to PPIs. However, these results were inconsistent with the results of the RCT conducted by Hamada et al.[10], which led us to perform the present study.”

**Comment 3:**

The authors said that “An intragastric pH of 5.4 or higher is required for blood coagulation to achieve haemostasis in peptic ulcer” and showed that vonoprazan provided the targeted pH. However, this was not only the ability of vonoprazan but also PPIs. Please revised those sentences, otherwise the reader maybe confused.

**Response:**

We appreciate your kind advice. As the reviewer pointed out, PPIs also have the same effect of increasing gastric pH as vonoprazan.

We have changed the sentence as follows (Page 9, Line 286 – Page10, Line 303).

“Treatment for ESD-induced artificial ulcers with gastric acid inhibitors is usually initiated on the date of ESD. However, post-ESD bleeding occurs most frequently within 24 hours after the procedure[5,35]. In the observational study analyzed here, vonoprazan was administered 1 day before ESD to achieve a high intragastric pH on the day of performing the technique, thereby facilitating effective hemostatic function. The RCT analyzed in the present study also involved the administration of vonoprazan in the same way.

Concerning gastric acid suppression, PPI administration can also achieve an intragastric pH of 5 or more, even when administered intravenously or orally. In the observational study, a combination of 2-day intravenous infusions of omeprazole and an oral intake of 20 mg of rabeprazole was adopted in the PPI group. A single infusion of omeprazole is known to quickly increase the intragastric pH > 4.25[20]. On the other hand, for the PPI group in the RCT, administration of oral lansoprazole 30 mg was started the day before ESD was conducted. The ratios of post-ESD bleeding in non-antithrombotic drug users were low and almost the same in the observational study and RCT (2.3% vs. 5.7%, respectively), suggesting that a sufficiently high intragastric pH was achieved by PPI administration on the day of ESD, to provide adequate prophylaxis against bleeding in non-high-risk patients.”

In addition, we have added the following sentences (Page 10 Line 308-312).

“However, esomeprazole and rabeprazole were better able to achieve an intragastric pH of 5 or higher after 7 days of administration (48.3% and 53.2%, respectively)[8]. Although initiating PPI therapy 7 days before ESD is difficult in everyday clinical practice, it might further reduce the risk of bleeding in antithrombotic drug users.”

**Comment 4:**

Please put the reference to this sentence “It remains unanswered whether the results obtained in RCTs are inconsistent with the facts observed in real-world clinical practice, which involves treating patients from diverse backgrounds.” In theory, this sentence may not true as the real-world data contains various type of bias and that is the reason for conducting RCT.

**Response:**

The relevant sections have been changed as follows (Page 10, Line 313-316).

“Because of various types of biases, patients with medical backgrounds that might affect the results were excluded from the study. However, it has been reported that the results of RCTs obtained in this way differ from those observed in real-world clinical practice[40-42].”

**Comment 5:**

The discussion part was too long and some sentences eg. “Keith and Constance reported that clinical studies evaluating the effects of alcohol treatment excluded patients with more severe alcohol disorders and those with low incomes and psychiatric problems”, etc. were not relevant to the present study. Please consider shortening the discussion part.

**Response:**

We reconsidered the discussion and shortened it overall.

In addition, we have changed the text in the parts that were pointed out to correspond to comment 4.

**Reviewer #2**

**Comment 1:**

The definition of “taking anti-thrombotic medication” lacks the timing and duration in relation to the procedure i.e. were the medications taken throughout periprocedural period or were they stopped at any point prior to the procedure.

**Response:**

We have added the withdrawal schedule for each antithrombotic drug in Figure 3.

The changes are as follows (Page 5, Line 112–113).

“The withdrawal schedules for each antithrombotic drug in the observational study are shown in Fig 3.”

We have changed the sentences in the introduction as follows (Page 2, Line 23-26).

“A meta-analysis revealed that antithrombotic drug users have a significantly increased risk of post-ESD bleeding than non-users, regardless of whether they suspended the treatment perioperatively. However, the analysis only included studies in which PPIs were used to treat post-ESD ulcers[17].”

**Comment 2:**

Among 3 RCTs included, the other two studies had sample size calculated too. Please specify more elaborative reasons why the study by Hamada et al. was chosen over the other two.

**Response:**

In the other two studies, the primary outcome was not post-ESD bleeding, but the ulcer reduction rate. Therefore, we used the study by Hamada et al., in which the sample size was calculated using post-ESD bleeding as the primary outcome. To show this, the changes are as follows (Page 3, Line 85-87).

“We decided to use the RCT by Hamada et al., which set the primary endpoint as bleeding after ESD and calculated the planned sample size.”

**Comment 3:**

Since Vonoprazan requires shorter onset of action and shorter duration to reach its steady level, administering oral PPI one day prior to the ESD procedures may not be adequate time for bleeding prophylaxis. This limitation should be discussed.

**Response:**

We appreciate your kind advice. As the reviewer pointed out, PPIs also have the same effect of increasing gastric pH as vonoprazan.

We have changed the sentence as follows (Page 9, Line 286-Page10, Line 303).

“Treatment for ESD-induced artificial ulcers with gastric acid inhibitors is usually initiated on the date of ESD. However, post-ESD bleeding occurs most frequently within 24 hours after the procedure[5,35]. In the observational study analyzed here, vonoprazan was administered 1 day before ESD to achieve a high intragastric pH on the day of performing the technique, thereby facilitating effective hemostatic function. The RCT analyzed in the present study also involved the administration of vonoprazan in the same way.

Concerning gastric acid suppression, PPI administration can also achieve an intragastric pH of 5 or more, even when administered intravenously or orally. In the observational study, a combination of 2-day intravenous infusions of omeprazole and an oral intake of 20 mg of rabeprazole was adopted in the PPI group. A single infusion of omeprazole is known to quickly increase the intragastric pH > 4.25[20]. On the other hand, for the PPI group in the RCT, administration of oral lansoprazole 30 mg was started the day before ESD was conducted. The ratios of post-ESD bleeding in non-antithrombotic drug users were low and almost the same in the observational study and RCT (2.3% vs. 5.7%, respectively), suggesting that a sufficiently high intragastric pH was achieved by PPI administration on the day of ESD, to provide adequate prophylaxis against bleeding in non-high-risk patients.”

In addition, we have added the following sentence (Page 10, Line 308-312).

“However, esomeprazole and rabeprazole were better able to achieve an intragastric pH of 5 or higher after 7 days of administration (48.3% and 53.2%, respectively)[8]. Although initiating PPI therapy 7 days before ESD is difficult in everyday clinical practice, it might further reduce the risk of bleeding in antithrombotic drug users.”

**Comment 4:**

As ESD with PPI prophylaxis performed before Vonoprazan became available were used as historical control, the bleeding outcome is inevitably confounded by endoscopists' experience which would be less than ESDs performed later (Vonoprazan group). Please discuss this limitation.

**Response:**

We have added the following sentences as a limitation of the study in the Discussion section (Page 10, Line 335-337).

“Third, although the same experienced endoscopists performed ESD in the PPI and vonoprazan groups in the observational study, the results may have been influenced by the endoscopists’ experience.”

**Comment 5:**

Statistically significant difference between each type of antithrombotic medications in each group (PPI vs Vonoprazan) should be presented in Table 5.

**Response:**

The risk differences and 95% confidence intervals between the vonoprazan and PPI groups by antithrombotic drug type are presented in Table 5 (Page 8).

**Comment 6:**

The authors rightfully stated that the risk of post-ESD bleeding differed between individual DOACs and other antithrombotic medication therefore, the differences between each antithrombotic regimen in both Vonoprazan and PPI group in this study needs to be further elaborated.

**Response:**

We have elaborated each antithrombotic regimen in both the vonoprazan and PPI groups (Page 7, Line 200-Page10, Line 222).

“Of the 86 patients in the vonoprazan group, 45 were on antiplatelet monotherapy, such as low-dose aspirin (LA)—32 were on LA, 11 on thienopridine, and 2 on other antiplatelet agents. Twenty-six patients received dual antiplatelet therapy (four received LA and thienopridine, seven received LA and other antiplatelet agents, and 15 received two other antiplatelet agents), of which two patients suffered post-ESD bleeding. Eight patients were on anticoagulant monotherapy (warfarin) and three were on direct oral anticoagulants (DOACs) (two on rivaroxaban and one on dabigatran), of which three and zero patients, respectively, experienced post-ESD bleeding. Three patients were on both, an antiplatelet agent and an anticoagulant (warfarin) (one on LA and warfarin, two on other antiplatelet agents and warfarin) and two patients were on both, an antiplatelet agent and an anticoagulant (DOAC) (one on other antiplatelet agents and apixaban, one on LA and dabigatran), of which zero and one patient, respectively, showed post-ESD bleeding. Of the 86 patients in the PPI group, 37 were on antiplatelet monotherapy (23 on LA, 10 on thienopridine, 4 on other antiplatelet agents) and 24 patients were on dual antiplatelet therapy (4 on LA and thienopridine, 7 on LA and other antiplatelet agents, 12 on two other antiplatelet agents), of which 3 and 7 patients, respectively, experienced post-ESD bleeding. Seventeen patients were on anticoagulant monotherapy (warfarin), of which three patients suffered post-ESD bleeding. Seven patients were on both, an antiplatelet agent and an anticoagulant (warfarin) (four on LA and warfarin, three on other antiplatelet agents and warfarin) and one patient was on both, an antiplatelet agent and an anticoagulant (DOAC) (LA and rivaroxaban), of which four and one patient, respectively, experienced post-ESD bleeding.”

In addition, we have added the following sentences in the Discussion section (page 9, Line 249-250, page 9, Line 260-264, and page 10, Line 299-303, respectively).

“Since only six DOAC users were included in this study, further studies are needed to determine the risk of post-ESD bleeding with specific anticoagulant drugs.”

“The post-ESD bleeding rate with the combined use of an antiplatelet and anticoagulant (warfarin or DOAC), which are presumed to pose the highest risk of bleeding, was lower in the vonoprazan group than in the PPI group (20.0% vs. 62.5%). The superiority of vonoprazan over PPI in preventing post-ESD bleeding may, therefore, be more pronounced in patients at a high risk of bleeding.”

“The ratios of post-ESD bleeding in non-antithrombotic drug users were low and almost the same in the observational study and RCT (2.3% vs. 5.7%, respectively), suggesting that a sufficiently high intragastric pH was achieved by PPI administration on the day of ESD, to provide adequate prophylaxis against bleeding in non-high-risk patients.”

**Comment 7:**

There is a few numerical errors e.g. Specific anti-thrombotic stratification is presented in Table 5, not 4

**Response:**

The relevant part has been corrected (Page 6, Line 184-185).

“The efficacy of vonoprazan compared with that of PPIs in preventing post-ESD bleeding is summarized in Table 4.”

**Comment 8:**

The difference in pharmacokinetic of vonoprazan and PPI should be moved to introduction.

**Response:**

The relevant part of the discussion has been moved to the Introduction ( Page 2, Line 8- Line 14) .

**Comment 9:**

There are a few grammatical and spelling errors. Please proofread the manuscript again.

**Response:**

We are sorry for the respective errors. These have been corrected.
